# Long non-coding RNA AC099850.4 correlates with advanced disease state and predicts worse prognosis in triple-negative breast cancer

**DOI:** 10.3389/fmed.2023.1149860

**Published:** 2023-08-31

**Authors:** Radhakrishnan Vishnubalaji, Nehad M. Alajez

**Affiliations:** ^1^Translational Cancer and Immunity Center (TCIC), Qatar Biomedical Research Institute (QBRI), Hamad Bin Khalifa University (HBKU), Qatar Foundation (QF), Doha, Qatar; ^2^College of Health and Life Sciences, Hamad Bin Khalifa University (HBKU), Qatar Foundation (QF), Doha, Qatar

**Keywords:** noncoding RNA, lncRNA, AC099850.4, biomarkers, triple negative breast cancer, prognosis

## Abstract

Our understanding of the function of long non-coding RNAs (lncRNAs) in health and disease states has evolved over the past decades due to the many advances in genome research. In the current study, we characterized the lncRNA transcriptome enriched in triple-negative breast cancer (TNBC, *n* = 42) and estrogen receptor (ER+, *n* = 42) breast cancer compared to normal breast tissue (*n* = 56). Given the aggressive nature of TNBC, our data revealed selective enrichment of 57 lncRNAs in TNBC. Among those, AC099850.4 lncRNA was chosen for further investigation where it exhibited elevated expression, which was further confirmed in a second TNBC cohort (*n* = 360) where its expression correlated with a worse prognosis. Network analysis of AC099850.4^high^ TNBC highlighted enrichment in functional categories indicative of cell cycle activation and mitosis. Ingenuity pathway analysis on the differentially expressed genes in AC099850.4^high^ TNBC revealed the activation of the canonical kinetochore metaphase signaling pathway, pyridoxal 5'-phosphate salvage pathway, and salvage pathways of pyrimidine ribonucleotides. Additionally, upstream regulator analysis predicted the activation of several upstream regulator networks including CKAP2L, FOXM1, RABL6, PCLAF, and MITF, while upstream regulator networks of TP53, NUPR1, TRPS1, and CDKN1A were suppressed. Interestingly, elevated expression of AC099850.4 correlated with worse short-term relapse-free survival (log-rank p = 0.01). Taken together, our data are the first to reveal AC099850.4 as an unfavorable prognostic marker in TNBC, associated with more aggressive clinicopathological features, and suggest its potential utilization as a prognostic biomarker and therapeutic target in TNBC.

## Introduction

Breast cancers represent a diverse group of cancers with different underlying biological features exhibiting differences in their clinical management, responses to treatment, and clinical outcomes (1). Recent advances in genomic research led to the BC classification of defined molecular subtypes, based on hormone receptor (HR), including estrogen receptor (ER) and progesterone receptor (PR), expression, as well as ERBB2 [also known as human epidermal growth factor receptor 2 (HER2)]

amplification, while tumors lacking overexpression of HR and lacking HER2 amplifications are referred to as triple-negative breast cancer (TNBC), comprising ~10–20% of all breast cancers. TNBC is oftentimes diagnosed at a younger age and has more aggressive clinicopathological features at presentation (larger tumor size, higher grade, and lymph node involvement) compared to other breast cancer subtypes. TNBC is also classified based on mRNA expression into four intrinsic subtypes: basal-like and immune suppressed (BLIS), immunomodulatory subtype (IM), mesenchymal-like subtype (MES), and luminal androgen receptor (LAR) subtype, with BLIS being the most aggressive subtype (2). While most of the research on breast cancer classification has focused on protein-coding mRNAs, the utilization of non-coding RNAs (ncRNAs), including miRNA and long non-coding RNAs (lncRNAs), is currently gaining momentum for breast cancer classification and as diagnostic and prognostic biomarkers (3–5). In our previous analysis, we identified 13 lncRNAs that were able to discriminate TNBC from normal breast tissue (3). A previous study by Huang et al. reported low NEAT1^low^ and MAL2^high^ to predict unfavorable outcomes in TNBC (6). In another study, Song et al. reported low-NEF lncRNA expression to correlate with poor prognosis in TNBC (7), thus corroborating a prognostic value for several lncRNA in TNBC.

lncRNAs represent a major class of ncRNAs with lengths exceeding 200 nucleotides and a lack of functional protein translation. lncRNAs can be divided into six different groups based on their genomic positions, subcellular localizations, and functions: (1) enhancer lncRNAs, (2) intronic lncRNAs, (3) antisense lncRNAs, (4) sense lncRNA, (5) intergenic lncRNA, and (6) bidirectional lncRNAs (8, 9). Increasing evidence has implicated lncRNAs in the onset and progression of various human cancers, through the regulation of key cellular processes, including proliferation, migration, invasion, and apoptosis at the transcriptional and post-transcriptional levels (10). Phase II/III clinical trials highlighted the potential use of RNA-based therapeutics, including antisense oligonucleotides (ASOs) and small interfering RNAs (siRNAs) to treat various human diseases (11).

Compelling data have implicated lncRNAs in regulating various biological processes, which could play oncogenic or tumor suppressor roles in breast cancer (12–15). Our data recently highlighted the prognostic and therapeutic functions of MALAT1 and LINC00511 in TNBC (16, 17).

In the current study, we characterized the differentially expressed lncRNAs in TNBC and ER^+^ breast cancers compared to normal breast tissues. Given the aggressive nature and lack of targeted therapies for TNBC, we subsequently aimed at identifying unique lncRNA transcripts expressed in TNBC, but not ER^+^ BC, which could potentially be used as prognostic biomarkers and therapeutic targets. Subsequently, we focused our study on AC099850.4 (alternatively named lnc-SKA2-1, AC099850.3, or ENSG00000265415), revealing AC099850.4 as a novel prognostic biomarker associated with unfavorable disease outcomes in TNBC. Comprehensive bioinformatics and network analysis revealed a plausible role of AC099850.4 in cell cycle regulation.

## Results

**Figure 1:**
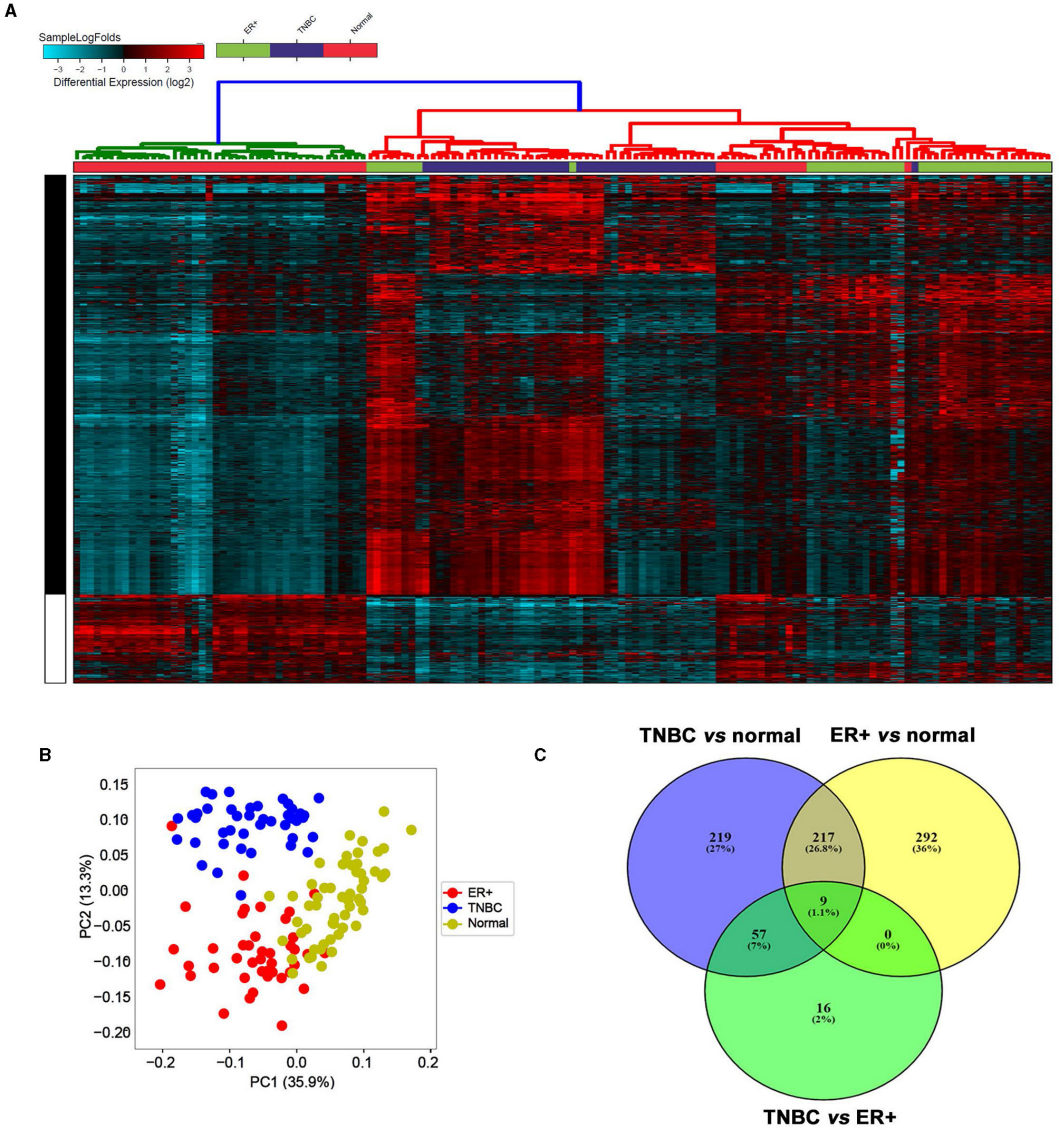
LncRNA transcriptional landscape in different breast cancer subtypes and normal breast tissue. **(A)** Hierarchical clustering of TNBC (*n* = 42), ER^+^ breast cancer (*n* = 42) and normal breast tissue (*n* = 56) based on differentially expressed lncRNAs. Each column represents one sample, and each row represents a single lncRNA. The expression level of each lncRNA (log2) is depicted according to the color scale. **(B)** Principal component analysis (PCA) for the lncRNA transcriptome of TNBC, ER^+^ breast cancer, and normal breast tissue. **(C)** Venn diagram depicting the overlap between upregulated lncRNAs in TNBC vs. normal, ER^+^ vs. normal, and TNBC vs. ER^+^.

To provide a global overview of the differentially expressed lncRNAs in different BC subtypes, transcriptomic data from 42 TNBC, 42 ER^+^HER2^−^ (referred to as ER^+^ throughout the article), and 56 normal breast tissues (NT) were pseudo-aligned to the GENCODE release (V33) reference genome using Kallisto. Data presented in Figure 1 revealed a distinct lncRNA expression profile for the indicated breast cancer molecular subtypes compared to NT (Figure 1A, Supplementary Table S1). Concordantly, PCA analysis revealed similar segregation of TNBC from ER^+^ and NT (Figure 1B). Our analysis revealed 226 lncRNAs that were upregulated in TNBC vs. NT and in ER^+^ vs. NT (Figure 1C). Interestingly, we identified 57 lncRNAs that were upregulated in TNBC vs. ER^+^ and in TNBC vs. NT, but not in ER^+^ vs. NT, suggesting their specific expression in TNBC (Figure 1C).

### AC099850.4 expression correlates with advanced tumor grade and worse prognosis

**Figure 2:**
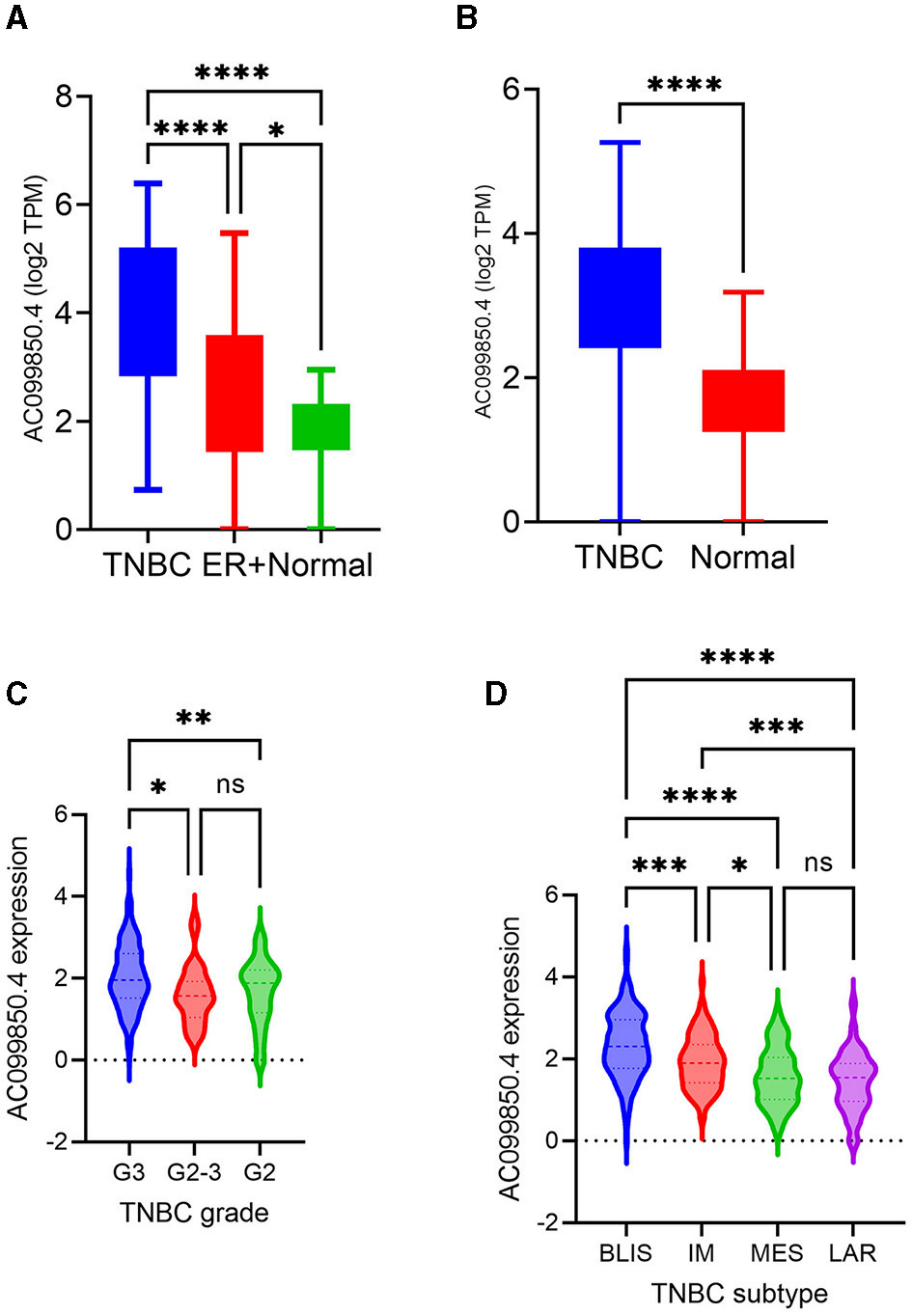
AC099850.4 expression correlated with advanced clinicopathological features of TNBC. **(A)** Box plots depicting the expression of AC099850.4 in TNBC (n = 42), ER^+^ BC (*n* = 42), and normal breast tissue (*n* = 56) from the PRJNA251383. **(B)** Expression of AC099850.4 in the validation cohort (TNBC = 360 vs. normal = 88) from PRJNA486023 **(C)** mRNA-based classification **(D)** TNBC. BLIS: basal-like immunosuppressed, MES: mesenchymal, IM: immunomodulatory, LAR: luminal androgen receptor. ^*^p < 0.05, ^**^p < 0.005, ^***^p < 0.0005 and ****p < 0.00005. Expression of AC099850.4 as a function of tumor grade.

Among the identified TNBC-enriched lncRNAs, AC099850.4 was chosen for further analysis since its expression was enriched in TNBC and has not been implicated in TNBC thus far. The expression AC099850.4 in TNBC, ER^+^, and NT is shown in Figure 2A. We subsequently confirmed the upregulated expression of AC099850.4 in a larger cohort of TNBC (*n* = 360) compared to normal (*n* = 88) exhibiting 2.2 fc, p(Adj) = 1.3 × 10^−30^, as shown in Figure 2B. Interestingly, we observed the highest expression of AC099850.4 in TNBC with advanced tumor grade (Figure 2C) and the BLIS TNBC subtype exhibiting the worst prognosis (18) (Figure 2D).

### Elevated expression of AC099850.4 correlates with the mitotic cell cycle in TNBC

**Figure 3:**
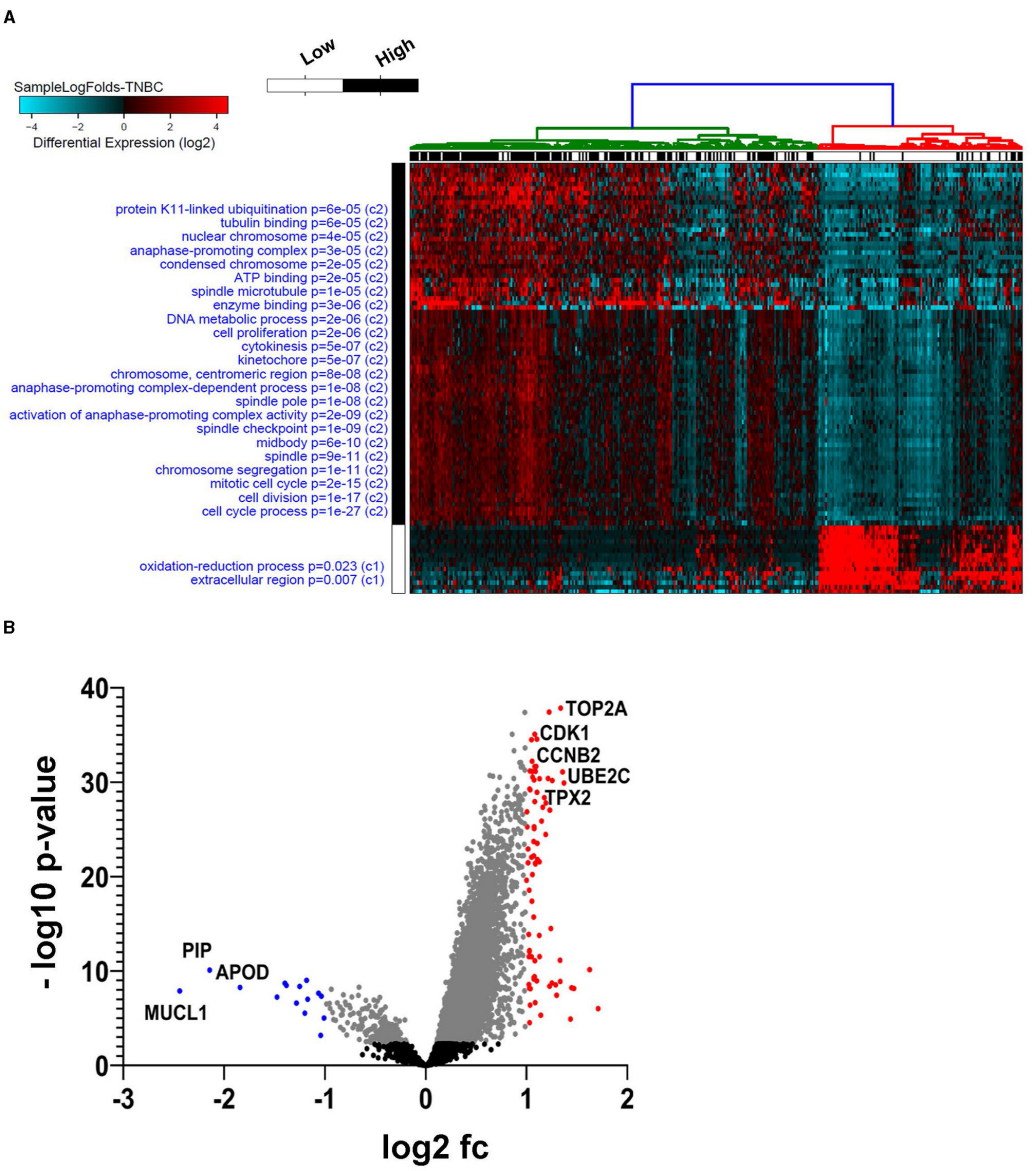
Functional enrichment in AC099850.4^high^ vs. AC099850.4^low^ TNBC. Expression data from 360 TNBC were grouped into “AC099850.4^high^” and “AC099850.4^low^” according to AC099850.4 median expression and were subjected to differential expression analysis. **(A)** Heatmap depicting the clustering of the AC099850.4^high^ vs. AC099850.4^low^ TNBC with the enriched gene ontology (GO) categories indicated on the left side and the corresponding enrichment *p*-value. **(B)** Volcano plot depicting the upregulated (red) and downregulated (blue) genes in AC099850.4^high^ vs. AC099850.4^low^ TNBC.

**Figure 4:**
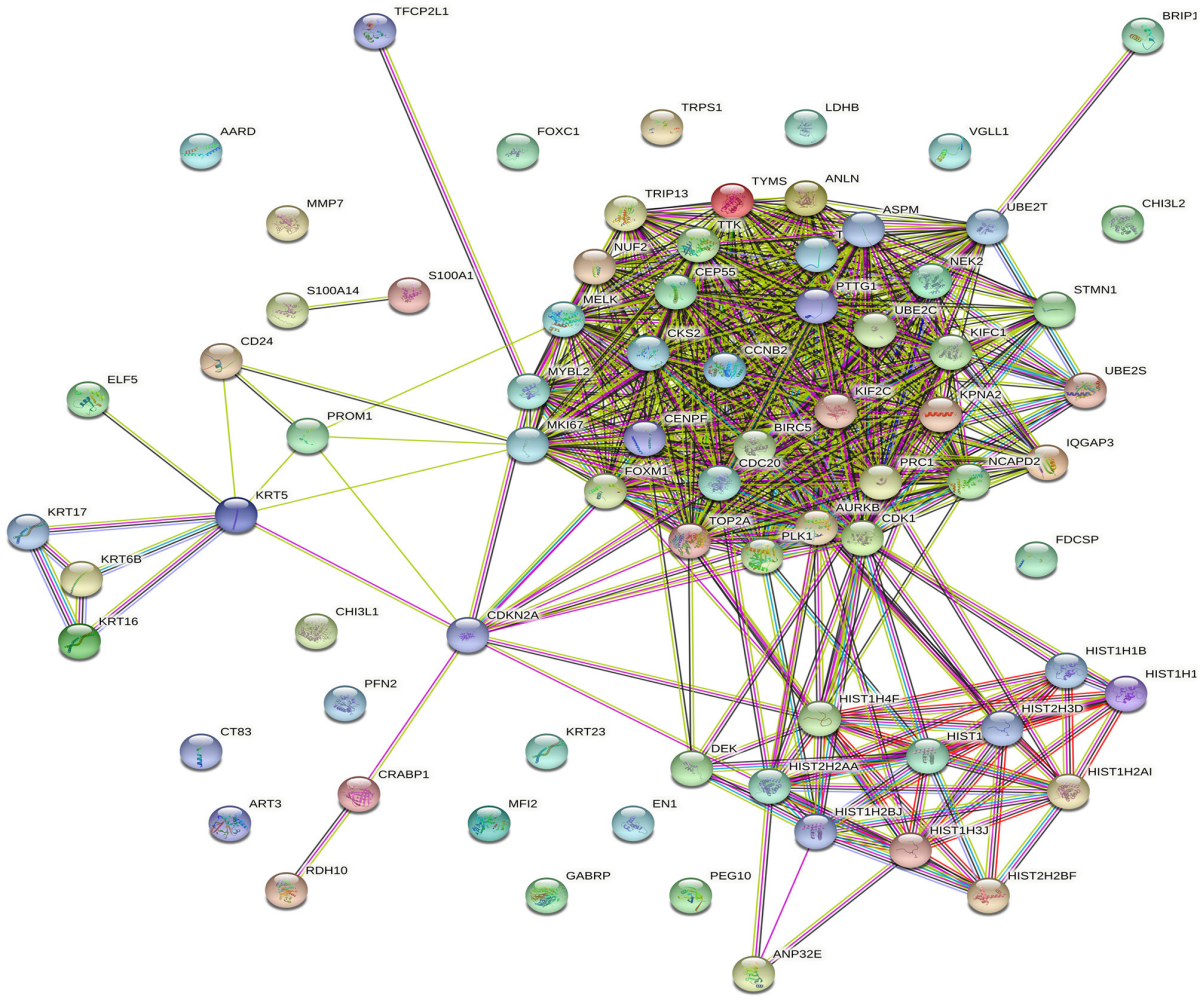
Protein-protein interaction (PPI) network analysis of upregulated genes in AC099850.4^high^ vs. AC099850.4^low^ TNBC. PPI network based on STRING analysis of upregulated genes in AC099850.4^high^ vs. AC099850.4^low^. Network statistics: number of nodes: 76, number of edges: 585, expected number of edges: 79, average node degree: 15.4, avg. local clustering coefficient: 0.647, PPI enrichment *p*-value: < 1.0 × 10^−16^.

To better understand the role of AC099850.4 in driving TNBC, the cohort of 360 TNBC was grouped into AC099850.4^high^ (*n* = 180) and AC099850.4^low^ (*n* = 180). We subsequently analyzed the corresponding protein-coding transcriptome of the AC099850.4^high^ vs. AC099850.4^low^ using the GENCODE v33 reference genome. Our data revealed a remarkable difference in mRNA expression between the AC099850.4^high^ vs. AC099850.4^low^, with majority of functional enrichment being in categories indicative of proliferation and mitosis (Figure 3A). Differentially expressed genes in AC099850.4^high^ are illustrated as volcano plot (Figure 3B). Protein–protein interaction (PPI) analysis on the upregulated genes in AC099850.4^high^ vs. AC099850.4^low^ revealed strong network interaction with the highest enrichment in cell cycle-related processes, where the expression of cell cycle regulators (TRIP13, MYBL2, BRIP1, UBE2S, ANLN, NUF2, CCNB2, MELK, PLK1, TPX2, BIRC5, AURKB, TYMS, NCAPD2, FOXM1, UBE2C, IQGAP3, CENPF, NEK2, ASPM, MKI67, TTK, CEP55, KIF2C, CDC20, CKS2, PTTG1, PRC1, CDK1, KIFC1, STMN1, TOP2A, and CDKN2A) was enriched in AC099850.4^high^ (Figure 4).

### Ingenuity pathway analysis of differentially expressed genes in AC099850.4^*high*^ vs. AC099850.4^*low*^ TNBC

**Figure 5:**
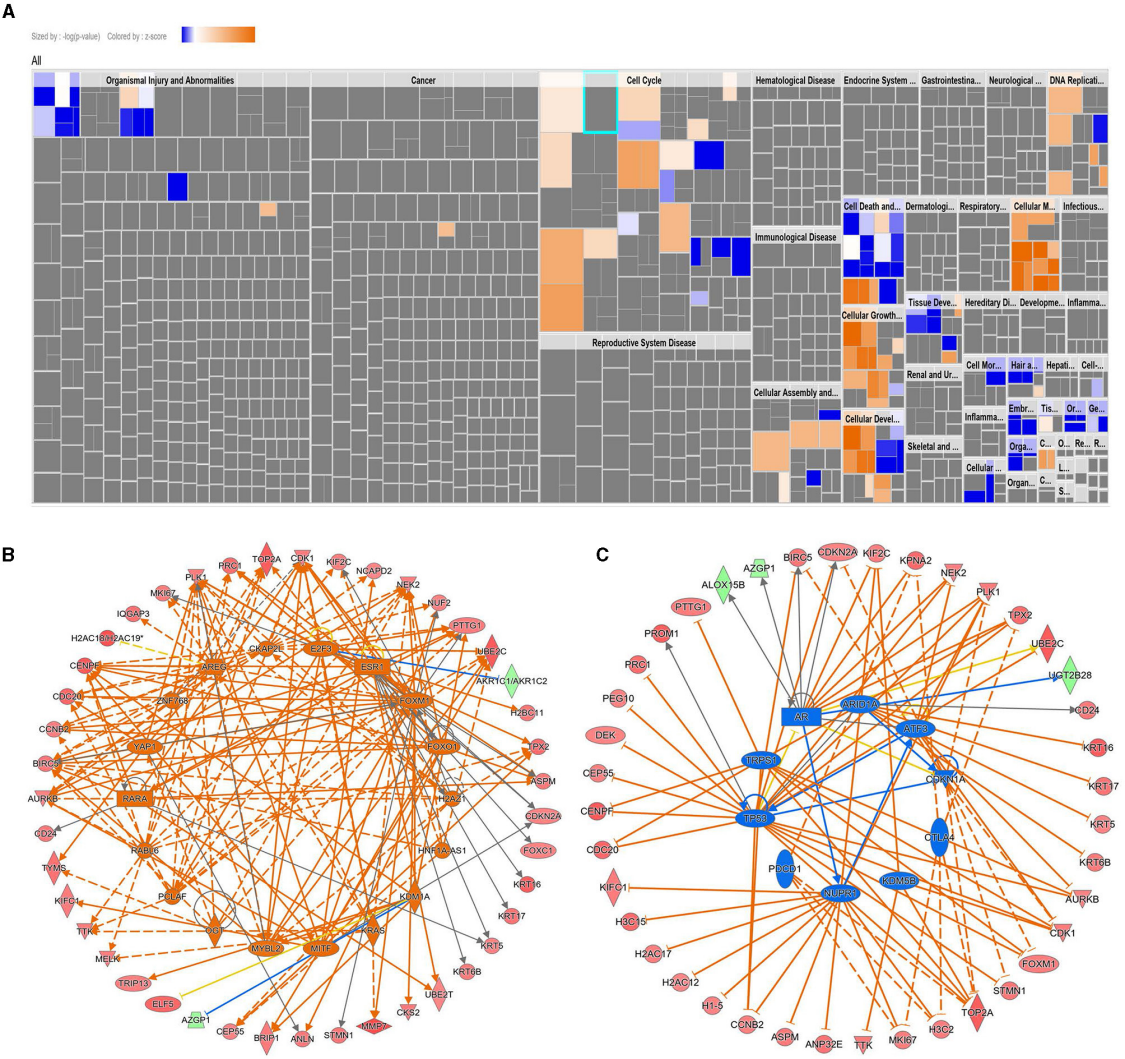
Ingenuity pathway analysis of differentially expressed genes in AC099850.4^high^ vs. AC099850.4^low^ TNBC. **(A)** Tree map (hierarchical heatmap) depicting affected functional categories based on differentially expressed genes in AC099850.4^high^ vs. AC099850.4^low^ where the major boxes represent a category of diseases and functions. Upstream regulator analysis depicting activated **(B)** and inhibited **(C)** networks in AC099850.4^high^ vs. AC099850.4^low^ TNBC.

We subsequently used ingenuity pathway analysis to provide a better understanding of the enriched canonical, upstream regulator, and disease and function categories in AC099850.4^high^ TNBC. Canonical enrichment analysis identified activation of the kinetochore metaphase signaling pathway, pyridoxal 5'-phosphate salvage pathway, and salvage pathways of pyrimidine ribonucleotides in AC099850.4^high^ TNBC (Supplementary Table S2). Disease and function analysis identified enrichment in cell proliferation, cell movement, migration of cells, invasion of cells, cell viability, and colony formation (Figure 5A, Supplementary Table S3). Upstream regulator analysis identified enrichment in networks with predicted activation state of CKAP2L, FOXM1, RABL6, PCLAF, MITF, FOXO1, AREG, H2AZ1, E2F3, ESR1, RARA, ZNF768, KRAS, HNF1A-AS1, OGT, YAP1, KDM1A, and MYBL2 (Figure 5B, Supplementary Table S4). In contrary, TP53, NUPR1, TRPS1, CDKN1A, CTLA4, AR, KDM5B, ARID1A, ATF3, and PDCD1 were suppressed (Figure 5C, Supplementary Table S4). Taken together, our data suggested a strong correlation between AC099850.4 expression and mitotic cell cycle in clinical tumor specimens from TNBC patients.

### AC099850.4 is an unfavorable prognostic biomarker for TNBC relapse-free short-term survival

**Figure 6:**
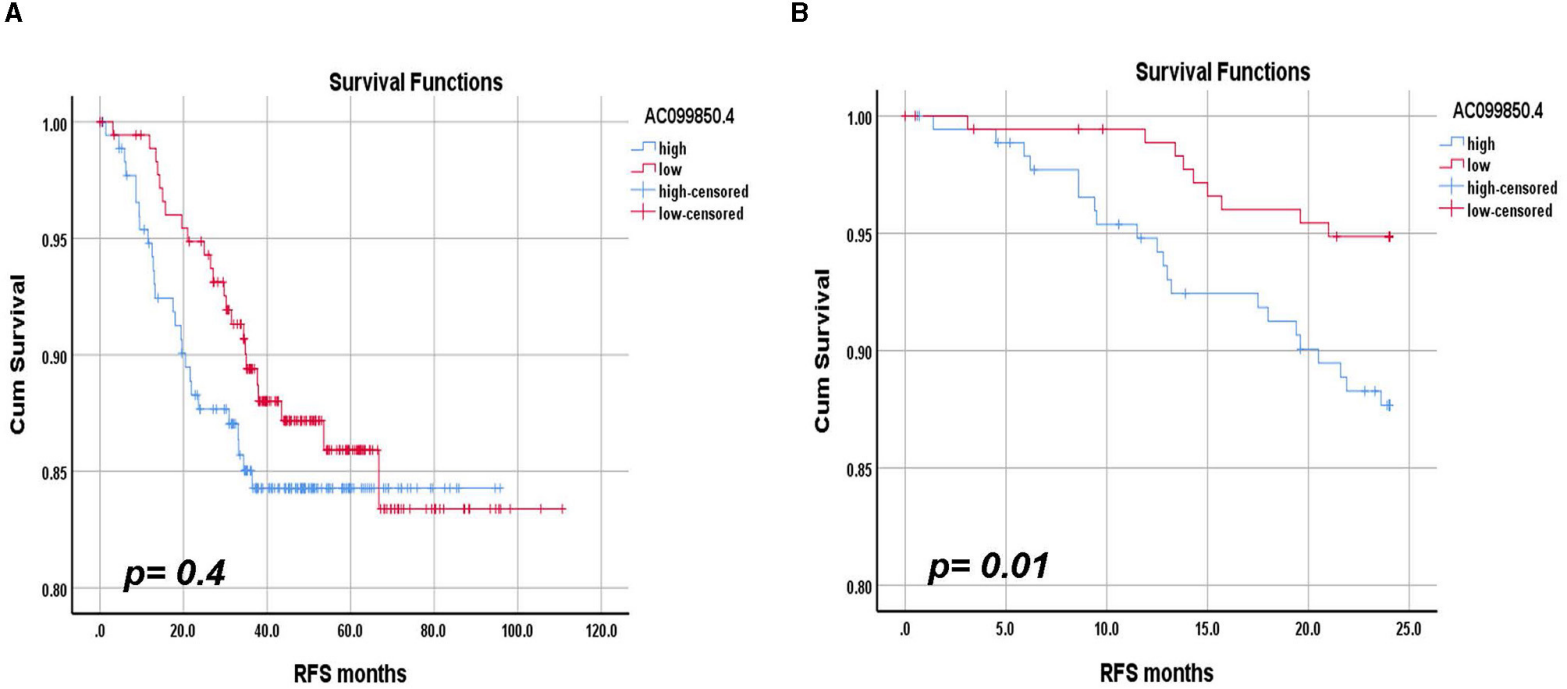
Relapse-free survival (RFS) analysis according to AC099850.4 expression. **(A)** Long-term RFS analysis in a cohort of 360 TNBC based on median AC099850.4 expression. **(B)** Short-term RFS analysis in a cohort of 360 TNBC based on median AC099850.4 expression. The log-rank test was used to compare groups.

We subsequently sought to assess the prognostic value of AC099850.4 in relation to RFS in TNBC. In that regard, we divided the 360 TNBC cohorts into AC099850.4^high^ and AC099850.4^low^ based on median AC099850.4 expression and performed the Kaplan–Meyer survival analysis. Interestingly, AC099850.4 expressed had a modest correlation with RFS in the long term (*log-rank p*-value = 0.4, Figure 6A). However, when we assessed the ability of AC099850.4 to predict short-term RFS (24 months), the high expression of AC099850.4 correlated with a worse prognosis (*log-rank p*-value = 0.01, Figure 6B). Those data highlighted a role for AC099850.4 as an unfavorable prognostic biomarker for short-term RFS.

## Discussion

Understanding the biological roles of various lncRNAs has contributed to our knowledge of the functions of this class of epigenetic regulators in cancer. In the current study, we characterized the lncRNA transcriptome of TNBC and ER^+^ breast cancers and identified 57 lncRNAs that were upregulated in TNBC vs. ER^+^ and in TNBC vs. NT, but not in ER^+^ vs. NT, suggesting their restricted expression in TNBC. Of particular interest, we conducted a comprehensive investigation on the expression AC099850.4 in TNBC. Interestingly, the highest expression of AC099850.4 was observed in TNBC patients with advanced tumor grade and in the BLIS subtype, which is known to have the worst prognosis among different TNBC subtypes (18). Investigating the expression of AC099850.4 in a larger cohort of TNBC (*n* = 360) correlated higher expression of AC099850.4 and enriched functional categories indicative of cellular proliferation and mitosis.

More in-depth computational analyses using IPA revealed activation of several functional categories in AC099850.4^high^ TNBC, including the canonical kinetochore metaphase signaling pathway, pyridoxal 5'-phosphate salvage pathway, and salvage pathways of pyrimidine ribonucleotides. Additionally, upstream regulator analysis predicted activation of CKAP2L, FOXM1, RABL6, PCLAF, and MITF and suppression of TP53, NUPR1, TRPS1, and CDKN1A in AC099850.4^high^ TNBC. Nonetheless, our data highlighted AC099850.4 as an unfavorable prognostic biomarker predicting short-term TRFS in TNBC. In agreement with our data, AC099850.4 was recently identified among 8 lncRNA biomarker panels in head and neck squamous cell carcinoma (19). Similarly, the elevated expression of AC099850.4, an m6A-related lncRNA, was reported in patients with oral squamous cell carcinoma (20), and the elevated expression of AC099850.4 was also correlated with worse survival in lung cancer (21). Recently, AC099850.4 was reported to be highly expressed and correlated with a worse prognosis in non-small cell lung cancer (22). Similarly, a recent study on hepatocellular carcinoma (HCC), which included 374 HCC and 160 non-HCC samples, identified five immune-related lncRNA prognostic panels, including AC099850.3. Silencing of AC099850.3 inhibited HCC cell proliferation and migration and led to significant inhibition of PLK1, TTK, CDK1, and BULB1 cell cycle molecules and CD155 and PDL1 immune receptors (23). Numerous recent studies revealed intriguing aspects of AC099850.4 as immuno-autophagy-related lncRNA (24), epithelial-mesenchymal transition-related lncRNA (25), and cancer cell stemness-associated lncRNA (26) in HCC. Those reports further support an oncogenic role for AC099850.4 in various human cancers, which remains to be validated in TNBC.

While several studies implicated AC099850.4 in various other cancer types, our data are the first to implicate this lncRNA in TNBC prognosis. Our data suggest the potential use of AC099850.4 as a prognostic biomarker and therapeutic target in TNBC, which warrants further investigation.

## Conclusion

Our data are the first to identify AC099850.4 as a novel prognostic biomarker for TNBC, correlating with advanced disease stage and patient survival.

## Limitations of the study

Our data provide solid evidence implicating AC099850.4 as a prognostic biomarker in TNBC. One limitation of the current study is that the cohort we analyzed has only ER^+^ and TNBC, but none of the patients were HER2^+^; hence, the expression of AC099850.4 in HER2^+^ BC remains to be assessed. Although our study was initially based on patients' transcriptomic data, the potential to utilize this lncRNA for patient prognosis remains to be validated in multiple TNBC cohorts. The functional consequences of AC099850.4 depletion in TNBC cell models remain to be validated *in vitro*, and the potential use of RNA-based therapeutics to target AC099850.4 systemically remains also to be addressed *in vivo*. Our data highlighted multiple enriched GO and networks in AC099850.4^high^ vs. AC099850.4^low^ TNBC; however, the exact mechanism by which AC099850.4 exerts its biological functions and its interacting protein partners remains to be identified using biochemical approaches, such as comprehensive identification of RNA-binding proteins by mass spectrometry, ChIRP-MS (27).

## Materials and methods

### RNA-Seq data analysis and bioinformatics

Raw RNA sequencing data were retrieved from the sequence read archive (SRA) database under accession no. PRJNA251383, consisting of 42 TNBC, 42 ER^+^HER2^−^, and 56 normal breast tissue samples. The Kallisto index was constructed by creating a de Bruijn graph employing the GENCODE release (V33) reference transcriptome and 31 length k-mer. FASTQ files were subsequently pseudo-aligned to the generated index using KALLISTO 0.4.2.1, as previously described (3, 28). Normalization (TPM, transcript per million) was conducted using KALLISTO 0.4.2.1. A detailed description of the study subjects can be found in Ref. (29). Normalized expression data (TPM) were sequentially imported into AltAnalyze v.2.1.3 software for differential expression and PCA analysis using 2.0-fold change and adjusted cut-off *p-*value of < 0.05 (30). Low abundant transcripts (< 1.0 TPM raw expression value) were excluded from the analysis. The Benjamini–Hochberg method was used to adjust for the false discovery rate (FDR). The marker finder prediction was carried out as previously explained. PRJNA486023 (360 TNBC and 88 normal samples) was retrieved from the SRA databases using the SRA toolkit v2.9.2 as previously described (31, 32) and was mapped to GENCODE release (v33) as mentioned above and was used to confirm our findings. Detailed information on the study subjects in this validation cohort can be found in Jiang et al. (33).

### Protein-protein interaction and KEGG network analysis

Upregulated genes in AC099850.4^high^ TNBC (*n* = 180) were subject to PPI network analysis using the STRING (STRING v10.5) database to illustrate the interacting genes/proteins based on knowledge and predication as described before (34). KEGG pathway analysis was conducted using DAVID as described earlier (35).

### Gene set enrichment and modeling of gene interactions networks

Upregulated genes in AC099850.4^high^ were imported into the Ingenuity Pathway Analysis (IPA) software (Ingenuity Systems; http://www.ingenuity.com/) and were subjected to functional annotations and regulatory network analysis using upstream regulator analysis (URA), downstream effects analysis (DEA), mechanistic network (MN) and causal network analysis (CNA) prediction algorithm. IPA uses precision to predict functional regulatory networks from gene expression data and provides a significance score for each network according to the fit of the network to the set of focus genes in the database. The *p-*value is the negative log of P and represents the possibility of focus genes in the network being found together by chance.

### Survival and statistical analysis

The Kaplan–Meier survival analysis and plotting were conducted using IBM SPSS version 26 software. For survival analysis, patients were grouped into high or low based on the corresponding lncRNA median expression. The log-rank test was used to compare the outcome between expression groups. GraphPad Prism 9.0 software (San Diego, CA, USA) was used to compare the lncRNA expression as a function of tumor grade and LN status. An unpaired two-tailed *t*-test was used to compare two groups, while a one-way ANOVA was used to compare multiple groups. The Benjamini–Hochberg method was used to adjust for the false discovery rate (FDR). The *p*-value of < 0.05 was considered statistically significant.

## Data availability statement

The datasets presented in this study can be found in online repositories. The names of the repository/repositories and accession number(s) can be found in the article/Supplementary material.

## Author contributions

RV performed the experiments and manuscript writing. NA obtained funding, concept, design, data analysis, and finalized the manuscript. All authors contributed to the article and approved the submitted version.
